# Imaging features of toxicities associated with immune checkpoint inhibitors

**DOI:** 10.1016/j.ejro.2022.100434

**Published:** 2022-08-08

**Authors:** Babina Gosangi, Lacey McIntosh, Abhishek Keraliya, David Victor Kumar Irugu, Akshay Baheti, Ashish Khandelwal, Richard Thomas, Marta Braschi-Amirfarzan

**Affiliations:** a Department of Radiology and Biomedical Imaging, Yale School of Medicine, New Haven, CT, USA; b Department of Radiology, University of Massachusetts, Worcester, MA, USA; c Department of Radiology, Brigham and Women’s Hospital, Harvard Medical School, Boston, MA, USA; d Magnus Hospital, Hyderabad, India; e Tata Memorial Hospital, Mumbai, India; f Department of Radiology, Mayo Clinic, Rochester, MN, USA; g Department of Radiology, Lahey Health System, Burlington, MA, USA

**Keywords:** Immune check point inhibitors toxicity, Pneumonitis, Colitis, Pancreatitis, Hepatitis, ICI, Immune check point inhibitor, CTLA-4 inhibitor, Cytotoxic T-lymphocyte antigen- 4 inhibitor, PD-L1 inhibitor, programmed cell death ligand-1 inhibitor, PD-1 inhibitor, programmed cell death-1 inhibitor, irAE, immune-related adverse event, CTCAE, Common Terminology Criteria for Adverse Events, PFS, progression free survival, RRP, radiation recall pneumonitis, FDA, Food and Drug Administration, NSCLC, non-small cell lung cancer, OP, organizing pneumonia, NSIP, non-specific interstitial pneumonia, AIP, acute interstitial pneumonitis, ARDS, acute respiratory distress syndrome, LGE, late Gadolinium enhancement

## Abstract

The past decade has witnessed a change in landscape of cancer management with the advent of precision oncology. Immune checkpoint inhibitors (ICIs) have revolutionized cancer treatment and have played an important role in improving patient survival. While the patients are living longer, treatment with ICIs are sometimes associated with adverse effects, some of which could be fatal. Radiologists can play a crucial role by early identification of some of these adverse effects during restaging scans. Our paper focuses on the imaging features of commonly occurring ICI toxicities based on organ system.

## 1 Introduction

The discovery of immune checkpoint inhibitors (ICIs) brought a paradigm shift in cancer management. The first ICI approved by the US Food and Drug Administration (FDA) in 2011 was Ipilimumab to treat metastatic melanoma [1]. Based on their mechanism of action, three major types of ICIs have been identified: Cytotoxic T-lymphocyte antigen- 4 inhibitor (CTLA-4 inhibitor), programmed cell death-1 inhibitor (PD-1 inhibitor), and programmed cell death ligand-1 and 2 inhibitors (PD-L1 and PD-L2 inhibitors) [2], [3]. Currently, FDA-approved ICIs include Ipilimumab (CTLA-4 inhibitor); Nivolumab, Pembrolizumab, and Cemiplimab (PD-1 inhibitors); and Atezolizumab, Durvalumab, and Avelumab (PD-L1 inhibitors) [4]. ICIs have been mainly approved for the treatment of advanced solid and hematological malignancies [4]. Additionally, FDA has also approved ICIs as the first-line therapy for some cancers like microsatellite instability-high or mismatch repair deficient colorectal cancer. ICIs are used either as monotherapy or as combination therapy termed “dual ICIs” or in combination with chemotherapy, so-called “chemoimmunotherapy.”

While these newer treatment agents have improved patient survival, they can be associated with adverse effects, ranging from abnormal laboratory tests or asymptomatic imaging findings to severe cases that can result in death. The negative effects or complications are termed “immune-related adverse events (irAE).” The incidence rate of irAEs can range from 30− 66% [5]. Studies have shown that irAE development is associated with improved efficacy of ICI, improved patient survival, and enhanced response rates [6], [7]. A prior study by Toi et al. in patients with non-small cell lung cancer (NSCLC) treated with Nivolumab showed that patients who experienced irAEs had a more prolonged median progression-free survival (PFS) of 12 months while patients without irAEs had a shorter median PFS of 3.2 months [6]. Therefore, early identification of irAE is key to continuing ICI therapy while managing the adverse effects in their early stages. This can be accomplished by modifying the therapy or starting supportive or mitigation measures such as steroid administration.

To standardize the reporting of adverse effect from anticancer agents, including the irAEs, the National Cancer Institute (NCI) developed specific terminology termed the “Common Terminology Criteria for Adverse Events (CTCAE), which helps in grading the severity of the events into 5 categories, [8].” Grade 1 includes asymptomatic toxicity which can be visible on imaging or toxicity with mild symptoms which do not require treatment, while grade 5 includes very severe cases which result in death [8]. Please refer to Table 1 for CTCAE classification [8]. CTLA-4 inhibitors are associated with more severe irAEs compared to PD-1/PD-L1 inhibitors. In a prior study, CTLA-4 inhibitors showed a greater incidence of grade 3 or higher irAEs (33%) compared to PD-1 inhibitors (10%) [5]. The incidence of irAEs is higher with combined therapy when compared to monotherapy with ICIs.

**Table 1:** CTCAE (Common terminology criteria for adverse events) for grading of irAE (immune-related adverse events).

Grade	Clinical features	Treatment
Grade 1	Asymptomatic or mild symptoms	Observation
Grade 2	Moderate in severity, limiting instrumental activities of daily life	Low dose steroids, ICI maybe suspended temporarily when considered necessary
Grade 3	Severe symptoms limiting activities of daily life but not life threatening	High dose steroids with temporary or permanent suspension of ICI, hospitalization maybe indicated
Grade 4	Severe life-threatening consequences	High dose steroids with discontinuation of ICI, hospitalization is indicated
Grade 5	Death	N/A

ICI-immune check point inhibitor

Radiologists must be aware of commonly occurring irAEs, specific irAEs associated with certain ICIs, and general timeframes to the onset of irAEs after starting therapy in order to improve their detection on imaging. For the convenience of our readers, we have classified irAEs based on organ systems and described their important clinical features and key imaging findings.

## 2 Thoracic irAEs

### 2.1 Pneumonitis

Drug-related pneumonitis is one of the major adverse events in patients receiving immunotherapy [9]. Pneumonitis is focal or diffuse inflammation of the lung parenchyma and is a result of immune-mediated injury, oxidative stress, or cytotoxic response [10]. Pneumonitis is the most common adverse effect of immunotherapy with an incidence rate of 4.1–19% with PD-1/PD-L1 checkpoint inhibitors [11], [12]. The rate is higher when combination therapy with PD-1/PD-L1 inhibitors and CTLA-4 inhibitors is used [11]. The lungs respond to these insults through different mechanisms, which on imaging have the appearance of interstitial pneumonias. Pneumonitis should be suspected when a patient started on immunotherapy complains of shortness of breath, cough, or chest pain. After starting immunotherapy, the median time to onset of pneumonitis is 2.6 months (0.5–11.5 months) [13]. The median time to onset is shorter when combination therapy is used [11]. Some patients with pneumonitis are now successfully treated with steroids and are able to continue with immunotherapy [14], [15]. Severe cases can be life-threatening, require hospital admission with intubation, and may result in death [14], [15]. The mortality rate can be as high as 20% [15]. Imaging plays a key role in detecting and monitoring patients with pneumonitis.

**Fig. 1:**
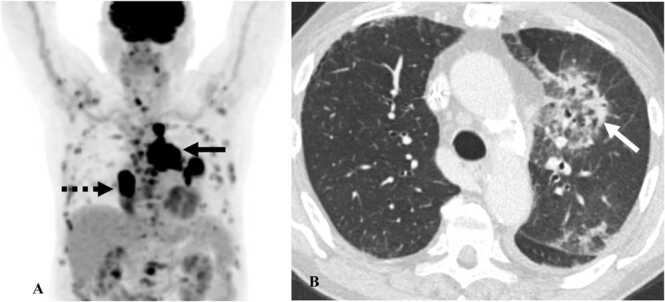
Immune-mediated pneumonitis in a 78-year-old man with small-cell lung cancer on Atezolizumab therapy. A. MIP image from PET/CT shows extensive hilar and mediastinal lymphadenopathy (black arrow) and right lower lobe lung mass (dashed black arrow). B. Contrast-enhanced axial chest CT image shows the development of new consolidation with central ground glass opacities (Atoll’s sign) in peribronchovascular distribution (white arrow) consistent with organizing pneumonia pattern of pneumonitis.

**Fig. 2:**
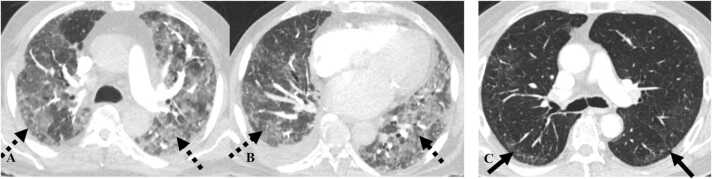
Immune-mediated pneumonitis. A&B. Contrast-enhanced axial chest CT images in a 63-year-old man with non-small-cell lung cancer on pembrolizumab show bilateral ground glass opacities with mild traction bronchiectasis (dashed black arrows) representing acute interstitial pneumonia or acute respiratory distress pattern of pneumonitis. C. Contrast-enhanced axial chest CT image in a 78-year-old man with melanoma on pembrolizumab shows peripheral reticulations with subpleural sparing (black arrows) consistent with NSIP pattern of pneumonitis.

On plain radiographs, pneumonitis often presents as bilateral airspace opacities. On CT, organizing pneumonia (OP) is the most commonly seen pattern of interstitial pneumonia associated with pneumonitis [13]. There are multifocal subpleural and peribronchovascular ground glass or consolidative opacities in the bilateral lungs [13]. A reversed halo appearance in which there is a central ground-glass opacity surrounded by peripheral consolidation can be seen with OP, called “Atoll’s sign” (Fig. 1) [13]. Non-specific interstitial pneumonia (NSIP) pattern is seen in some cases, demonstrating peripheral reticulations with ground-glass opacities or consolidations with sub-pleural sparing and traction bronchiectasis [13]. Hypersensitivity pneumonitis is characterized by upper lobe predominant ground-glass opacities with centrilobular nodules and lobular areas of air trapping [13]. Acute interstitial pneumonitis (AIP) pattern is rarely encountered, and the imaging features are similar to acute respiratory distress syndrome (ARDS) with bilateral ground glass and consolidative opacities in an apicobasal gradient [13]. Lower lobes show dense consolidations while the upper lobes have ground-glass opacities with traction bronchiectasis (Fig. 2) [13]. On PET/CT, pneumonitis presents as FDG-avid bilateral consolidations. In some rare cases, pneumonitis flare is seen [9]. Pneumonitis flare is the recurrence of pneumonitis after the initial episode of pneumonitis is successfully treated and steroid administration is discontinued [9]. This can happen with or without rechallenge with the offending drug. On imaging, pneumonitis flare presents with similar but more extensive opacities in the bilateral lungs and is more severe than the initial presentation [9].

### 2.2 Sarcoidosis-like reaction

Sarcoidosis-like reaction is most commonly seen with ipilimumab (5–7%) though cases have been reported with nivolumab and pembrolizumab (<0.5%) [16], as well as other targeted therapies. The median time to onset of sarcoidosis-like-reaction from initiation of immunotherapy is approximately 14 weeks, ranging from 3 weeks to 2 years [16]. Sarcoidosis-like-reaction can be challenging to diagnose as the imaging features overlap with features of the natural progression of cancer. However, sarcoidosis-like-reaction has a characteristic distribution and either improves or resolves over a period when immunotherapy is continued [16]. Though not routinely indicated, tissue biopsy remains the gold standard for distinguishing sarcoidosis-like-reaction from tumor progression [17]. Steroid administration is reserved for symptomatic cases when patients present with fatigue, fever, or shortness of breath. Mediastinal nodes are the most common site of involvement, followed by skin, lungs, extrathoracic nodes, spleen, CNS, eye, and bone.

**Fig. 3:**
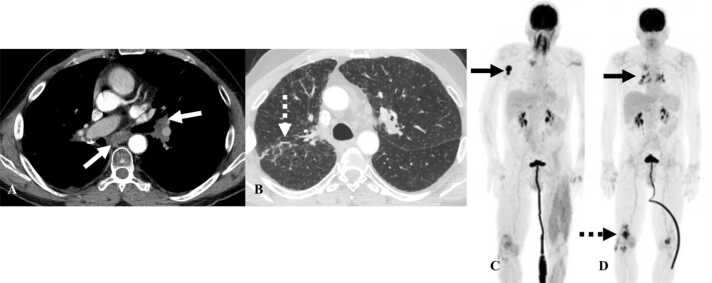
Sarcoidosis-like reaction. A&B. Contrast-enhanced axial chest CT images in a 57-year-old man with lung cancer 9 months after initiation of pembrolizumab show mediastinal and hilar lymphadenopathy (white arrows) and innumerable perilymphatic nodules on lung windows (dashed white arrow). C. Reconstructed MIP image from PET/CT in a 54-year-old man with melanoma shows FDG-avid right axillary lymphadenopathy (black arrow), consistent with biopsy proven melanoma. D. Reconstructed MIP image from PET/CT obtained 6 months after the initiation of Nivolumab and Ipilimumab shows resolution of sites of melanoma but new FDG-avid mediastinal and hilar lymphadenopathy (black arrow) consistent with sarcoidosis-like-reaction. Also note inflammatory arthritis in the bilateral knees (dashed black arrow).

On CT, the most classic pattern consists of mediastinal and hilar lymphadenopathy with or without bilateral pulmonary nodules (Fig. 3) [18]. Ground glass opacities are uncommon but can be seen [18]. The abdomen is the most common extrathoracic site of involvement. Splenic involvement presents as splenomegaly with small hypodense lesions in the spleen [19]. In rare cases co-existing intra-abdominal lymphadenopathy may be seen. Interstitial nephritis presents with striated nephrogram on CT with alternating hypodense and hyperdense areas [20]. Pituitary involvement is best diagnosed on MRI with diffuse enlargement of the pituitary gland and infundibulum with intense enhancement on post contrast images and restriction on diffuse weighted imaging [21]. On PET/CT, there is FDG uptake in the involved lymph nodes and tissues (Fig. 3).

### 2.3 Radiation recall pneumonitis

**Fig. 4:**
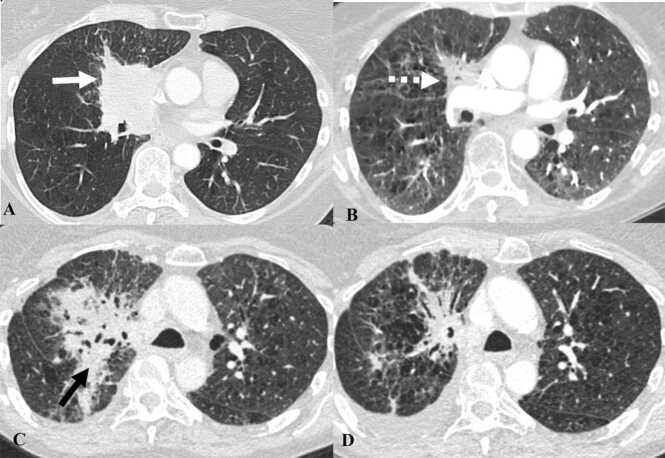
Immune-mediated radiation recall pneumonitis in a 65-year-old man with non-small cell lung cancer treated with Durvalumab therapy. Contrast-enhanced axial chest CT images demonstrate the following: A. Right perihilar mass consistent with biopsy proven non-small cell lung cancer (white arrow). B. Patient was treated with radiation and chemotherapy (cistplatin+etoposide) after which the primary tumor decreased in size (dashed white arrow). C. The patient was started on Durvalumab therapy after 2 years with worsening consolidation at the site of prior radiation in the right perihilar region (black arrow), suspicious for radiation recall pneumonitis. D. Durvalumab was stopped, and the patient was placed on steroids after which the pneumonitis decreased.

Radiation recall pneumonitis (RRP) is a very rare complication known to occur with conventional anticancer agents, such as gemcitabine, targeted therapy and immunotherapy [22]. Radiation induced inflammatory changes in the lung subside around 6–9 months after radiotherapy [22]. In RRP, new inflammatory changes occur in the previously radiated lung field after a prolonged quiescent stage triggered by the administration of systemic therapy [23]. Prior case reports have revealed that the time interval from prior radiotherapy to the onset of RRP in patients who received immunotherapy averaged about 2 years [24]. On CT, new ground glass or consolidative opacities are seen at or around an area of prior radiation which has remained quiescent for several months or years after radiotherapy (Fig. 4) [22]. Organizing pneumonia pattern is the most common pattern of pneumonitis associated with RRP [22]. Corticosteroids are the mainstay of treatment and angiotensin converting enzyme inhibitor is used for symptomatic relief [25], [26].

## 3 Cardiovascular irAE

### 3.1 Myocarditis

The incidence of myocarditis is low and ranges from 0.04% to 1.14% however carries a very high mortality rate ranging from 25% to 50% [27], [28], [29], [30]. The average duration to the onset of myocarditis from the start of therapy is 30–60 days [27], [28]. Though the exact mechanism of myocarditis has yet to be determined, the proposed mechanism is a shared antigen between the tumor and the myocardium leading to molecular mimicry resulting in T-cell infiltration of the myocardium [27]. Myocarditis can be asymptomatic with abnormal electrocardiogram and elevated cardiac biomarkers (Troponin I and B-type natriuretic peptide when associated with heart failure) [28], [31]. It usually manifests with chest pain, dyspnea, fatigue, or palpitations when symptomatic. In more severe cases, arrhythmia, heart block, or ventricular tachycardia can occur and result in cardiogenic shock [27], [28]. Endomyocardial biopsy establishes the diagnosis [32]. It is treated by cessation of immunotherapy and administering steroids.

**Fig. 5:**
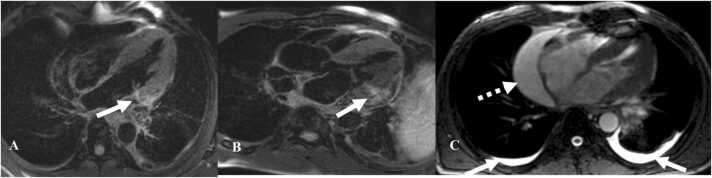
Immune-mediated myocarditis in a 67-year-old man with non-small cell lung cancer treated with Durvalumab. A&B. Post contrast axial MRI images show enhancement of the lateral and basal part of the left ventricle (white arrows) C. T2WI shows pericardial effusion along the right heart border (dashed white arrow) and small bilateral pleural effusions (white arrows).

Cardiac MRI is the imaging modality of choice for myocarditis. T2 hyperintensity in the myocardium and late gadolinium enhancement (LGE) is characteristic of myocarditis [33]. Other supportive findings are the presence of pericardial effusion or abnormal signal intensity in the pericardium on T2 images or the presence of LGE (Fig. 5) [33]. Regional or global wall motion abnormalities can be seen with decreased left ventricular ejection fraction in some cases [33]. On cardiac protocol PET/CT, FDG-avidity localized to the myocardium is usually diffuse and patchy in distribution.

### 3.2 Pericardial disease

**Fig. 6:**
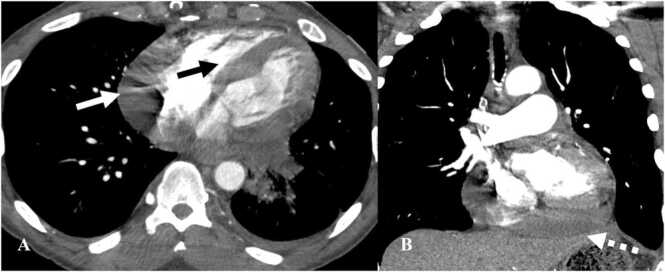
Immune-mediated pericarditis in a 67-year-old man with non-small cell lung cancer treated with Durvalumab. A. Contrast-enhanced axial chest CT image shows pericardial effusion along the right heart border (white arrow). Note the straightening of interventricular septum which can be seen with tamponade (black arrow). B. Coronal image in the same patient shows a large pericardial effusion at the base of the heart (dashed white arrow).

Pericardial involvement is a less frequently seen adverse effect of ICI therapy [34] and may manifest with pericarditis, pericardial effusion, or tamponade [34]. Pericarditis is seen as enhancement of the pericardium on contrast-enhanced CT. Pericardial effusions are typical, with variable amount of fluid in the pericardial space. When a large amount of fluid accumulates in the pericardium over a short amount of time, it can result in tamponade (Fig. 6). Tamponade is increased pressure within the pericardium associated with the collapse of the right ventricle, bowing of the interventricular septum, enlargement of superior venacava, inferior venacava (IVC), hepatic, and renal veins, reflux of contrast into the IVC or azygos vein. Tamponade is an emergency, and treatment is immediate pericardiocentesis.

### 3.3 Vasculitis

**Fig. 7:**
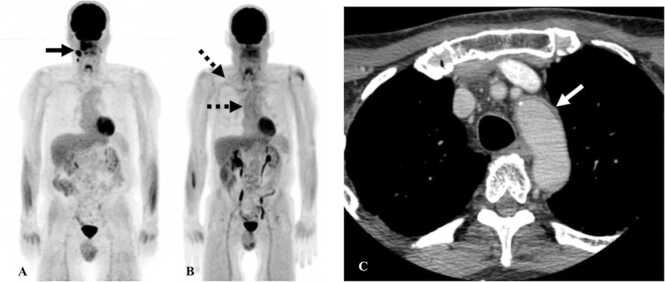
Immune-mediated vasculitis in a 67-year-old man with squamous cell cancer of the oropharynx on Nivolumab. A. Reconstructed MIP image from PET/CT shows FDG-avid lesion in the floor of mouth with lymphadenopathy (black arrow). B. Reconstructed MIP image from PET/CT obtained 6 months after initiating Nivolumab therapy shows resolution of the lesion with new FDG uptake along the walls of the aorta and subclavian arteries consistent with vasculitis (black dashed arrow). C. Contrast-enhanced CT of the neck from the same day shows wall thickening of the arch of aorta (white arrow).

Vasculitis is a rare complication of immunotherapy with less than 1% incidence rate [35]. Large-vessel vasculitis is more common with features of giant-cell arteritis (GCA) [36], [37]. CT or MR angiography is the diagnostic modality of choice to diagnose vasculitis. There is diffuse circumferential thickening of the vessel wall with mural enhancement or thrombi (Fig. 7). When vasculitis involves the aorta, it can extend into the origin of vessels arising from the aorta such as the subclavian artery, left common carotid artery, superior mesenteric artery, or renal arteries [38]. On PET/CT, FDG-avidity is seen along the wall of the vessel. Less commonly, vasculitis can occur in end-organ arteries causing damage to the involved organ. Vasculitis involving arteries of the jejunum can present with imaging features of occlusion of branch of superior mesenteric artery (SMA) without a true SMA occlusion. For instance, on CT there can be focal dilatation of the jejunum with the enhancement of the mucosa and wall thickening [39]. Vasculitis is usually treated with steroids.

## 4 Gastrointestinal irAE

### 4.1 Colitis

The incidence of colitis ranges from 8-22% (3% with PD1 inhibitors, 6% with CTLA-4 inhibitors, and 9% with combined therapy) [40], [41]. The median time to onset of colitis from the initiation of immunotherapy is approximately 8 weeks (2–4 months) [42]. Patients present with pain abdomen and diarrhea. Diffuse colitis is treated with steroids alone, but focal colitis is treated with steroids and antibiotics. Infliximab and vedolizumab are used in steroid refractory cases [43], [44].

**Fig. 8:**
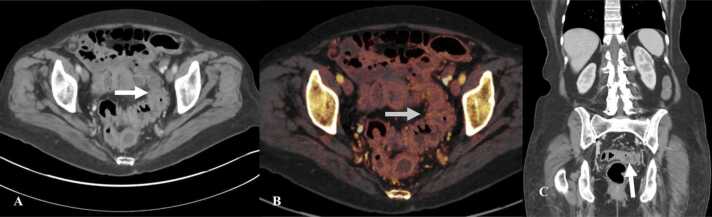
Immune-mediated colitis in a 73-year-old woman with renal cell cancer on Nivolumab. A. Contast-enhanced axial CT of the abdomen and pelvis shows wall thickening and enhancement of the descending and sigmoid colon (arrow) with adjacent fat stranding. B. Color overlay image on dual energy CT shows enhancement in the mucosa of sigmoid colon (arrow). C. Coronal image of the abdomen and pelvis shows wall thickening and enhancement of the mucosa of sigmoid colon (arrow).

Two different types of imaging features have been described on CT: diffuse colitis and segmental colitis [42]. Diffuse colitis is characterized by the involvement of a long segment of colon with mucosal enhancement, bowel wall thickening, fluid in the bowel lumen, and mesenteric vessels engorgement (Fig. 8) [42]. Focal colitis is seen in a bowel segment with pre-existing diverticulosis [42]. In focal colitis there is a short segment bowel wall thickening with mucosal enhancement and pericolonic fat stranding [42]. On PET/CT there is FDG uptake within the mucosa of the involved segment of the colon.

### 4.2 Gastroenteritis

Gastritis and enteritis have rarely been reported and occur less frequently than colitis, with few cases described. Gastritis can occur synchronously with Helicobacter or Cytomegalovirus virus infection [45]. The median time to onset of gastritis is around 4–9 months after initiation of ICI therapy [46], [47]. Enteritis has been reported to occur within one month of starting immunotherapy [48]. Patients may complain of pain abdomen, vomiting, nausea, or weight loss. Treatment includes cessation of the offending ICI with steroid administration [46].

On CT imaging, gastritis is seen as diffuse gastric wall thickening with perigastric fat stranding [47]. Endoscopy can confirm the diagnosis and shows mucosal erythema with ulcers. Enteritis is characterized by diffuse wall thickening of the small bowel with mucosal enhancement, distension of the lumen with fluid, and adjacent fat stranding [48]. Diffusely increased FDG-uptake is seen in the walls of the involved bowel segments.

## 5 Hepatopancreaticobiliary irAE

### 5.1 Hepatitis

Hepatotoxicity occurs in 1–17% of patients on ICI therapy [49]. The incidence rate is higher with combination therapy. The median time to onset of hepatotoxicity after starting ICI therapy is about 6–14 weeks [50]. The majority of cases present with asymptomatic elevation of liver enzymes (alanine aminotransferase and aspartate aminotransferase). In symptomatic patients, clinical features include pain in the right upper quadrant, jaundice, pruritis, abdominal distension due to ascites, confusion progressing to coma in hepatic encephalopathy. Corticosteroids are the first line therapy [51]. Azathioprine and Tacrolimus are reserved for patients who do not respond to steroids [52].

**Fig. 9:**
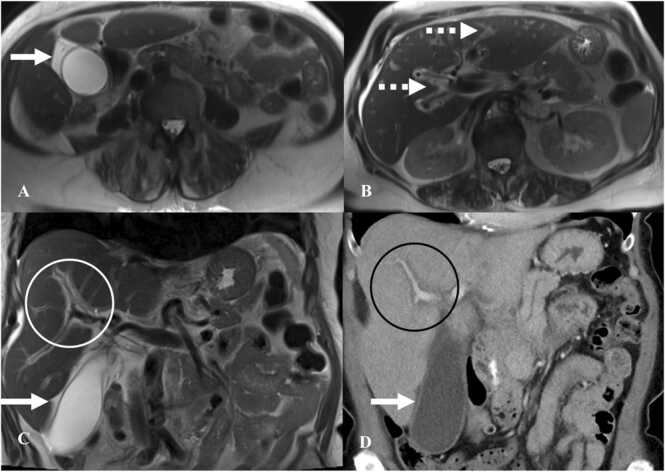
Immune-mediated hepatitis in a 68-year-old man with melanoma on Pembrolizumab. A&B. Axial T2W images of MRI of the abdomen show gallbladder wall edema (white arrow) and periportal edema (dashed white arrow). C. Coronal T2W MRI abdomen image shows periportal edema (white circle) and gallbladder wall edema (arrow). D. Coronal image on contrast-enhanced CT of the abdomen shows periportal edema (black circle) with gallbladder wall edema (arrow). Also note diffuse hypoattenuation of the liver.

Imaging features of immunotherapy-related hepatitis are identical to acute hepatitis. Ultrasound shows hepatomegaly and diffusely hypoechoic liver parenchyma with hyperechoic portal triads/periportal edema (“starry sky” appearance) often accompanied by gallbladder wall thickening. CT shows hepatomegaly with hypoattenuation of the hepatic parenchyma with periportal edema, as evidenced by decreased attenuation around the portal system (Fig. 9) [50]. Co-existing periportal lymphadenopathy can also be seen. On MRI, there is increased parenchymal signal intensity on T2-weighted images with increased signal intensity around the portal system consistent with periportal edema with delayed periportal enhancement (Fig. 9).

### 5.2 Pancreatitis

The incidence rate of ICI-related pancreatitis ranges from 2-8% (2% with CTLA-4 inhibitor, 4% with PD-1/PD-L1 inhibitor, and 8% with combination therapy) [53]. The median time from immunotherapy initiation to the onset of pancreatitis ranges from 2 to 5 months (69 days for CTLA-4 inhibitor, 146 days for PD-1/PD-L1 inhibitor, and 110 days for combination therapy) [53]. The majority of patients are asymptomatic with elevation of serum lipase levels [53]. Symptomatic patients present with epigastric pain, nausea, vomiting, fever, and diarrhea. ICI-related pancreatitis is treated by cessation of the offending ICI therapy, steroid administration, and fluid resuscitation [53].

**Fig. 10:**
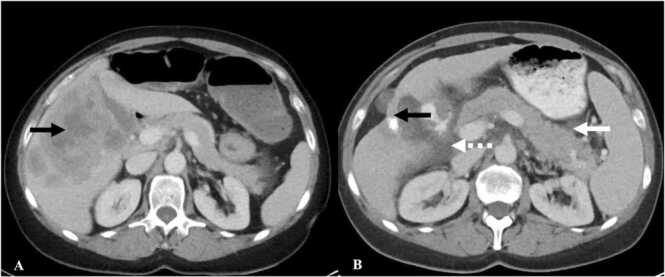
Immune-mediated pancreatitis in a 55-year-old man with melanoma on Pembrolizumab. A. Contrast-enhanced axial CT abdomen image shows numerous hepatic metastases (black arrow) with normal appearing pancreas. B. Contrast-enhanced axial CT abdomen image after the initiation of Pembrolizumab shows decrease in the hepatic metastases with liver capsule retraction (black arrow). Note that the pancreas is now bulky with peripancreatic stranding (white arrow) and fluid in the pancreaticoduodenal groove (dashed white arrow) suggesting pancreatitis.

On CT, diffuse pancreatitis presents as diffuse enlargement of the pancreas with heterogenous enhancement, and peripancreatic stranding (Fig. 10) [54]. In focal pancreatitis there is segmental hypoenhancement with peripancreatic stranding [54]. Complications such as pancreatic necrosis and hemorrhage are rare but reported. Pancreatic atrophy was seen as a chronic sequela in one study [54]. Increased FDG-avidity in and around the pancreas can be seen on PET/CT.

### 5.3 Cholangitis

The incidence of ICI-related cholangitis is low, for example, 3.3–6.8% with Nivolumab [55], [56]. The median duration to the onset of cholangitis is approximately 11–22 weeks [56]. Abdominal pain is the most common symptom, followed by fever and jaundice [56]. ERCP-guided biopsy helps in establishing the diagnosis [56]. Corticosteroids are the mainstay of treatment but the response rate to steroid administration is low at about 11.5% [56].

**Fig. 11:**
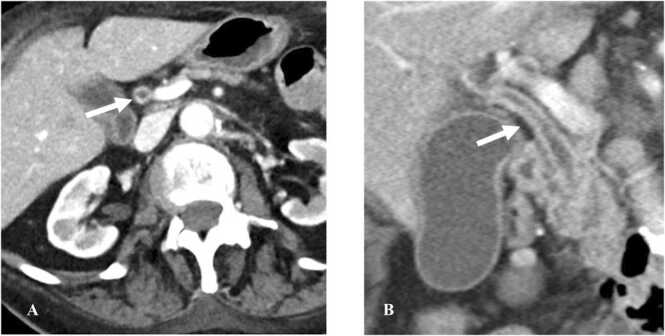
Immune-mediated cholangitis in a 72-year-old woman with melanoma on Pembrolizumab presenting with jaundice and elevated liver enzymes. A. Contrast-enhanced axial abdomen CT image shows enhancement in the wall of common bile duct (CBD) (arrow). B. Contrast-enhanced coronal abdomen CT image shows diffuse enhancement of the wall of CBD (arrow). Findings are consistent with cholangitis.

CT shows variable intrahepatic biliary duct dilatation with diffusely thickened and enhancing walls of the bile duct (Fig. 11) [55]. MRI also shows intrahepatic biliary ductal dilatation with enhancement of the bile duct walls on post contrast imaging [57]. Pruned tree appearance of the bile ducts is seen in advanced cases as the condition progresses from extrahepatic bile ducts to intrahepatic bile ducts [57].

### 5.4 Cholecystitis

The incidence of ICI-related cholecystitis is low at 0.6% [58]. The median time to onset of cholecystitis is 6 months (0.1–31 months) [58]. Common symptoms are abdominal pain, nausea, diarrhea, and fever [58]. Approximately 20% of cases resolve with steroid administration while 80% cases are treated with percutaneous drainage or cholecystectomy [58].

**Fig. 12:**
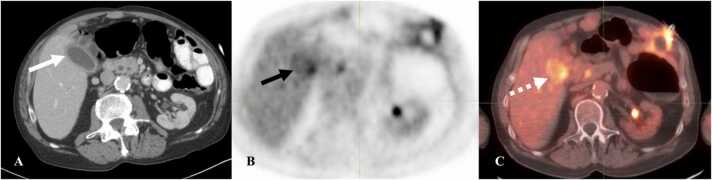
Immune-mediated cholecystitis in an 88-year-old woman with squamous cell cancer of the left tonsil on pembrolizumab. A. Contrast-enhanced axial CT abdomen image shows distended gallbladder with enhancing mucosa and pericholecystic fluid (white arrow) suspicious for cholecystitis. B. Axial PET/CT image shows uptake in the gallbladder wall (black arrow). C. Fused axial PET/CT image shows FDG uptake in the gallbladder wall suggesting cholecystitis (dashed white arrow).

On ultrasound, the gallbladder is distended with wall thickening, pericholecystic fluid and fat stranding. On CT, the gallbladder is distended with mucosal enhancement, enhancement of the adjacent liver parenchyma, gall bladder wall thickening with pericholecystic fluid and stranding (Fig. 12). PET/CT may show increased FDG uptake of the gallbladder wall.

## 6 Endocrine irAE

### 6.1 Hypophysitis

The incidence of hypophysitis is higher with CTLA-4 inhibitors compared to PD-1/PD-L1 inhibitors [59]. A meta-analysis by Filette et al. reported that the incidence of hypophysitis with CTLA-4 inhibitors is 5.6%, PD-1/PD-L1 inhibitors is 0.5%, and combination therapy with CTLA-4 inhibitor and PD-1 inhibitor is 10.5% [59]. The disproportionate higher incidence of hypophysitis with CTLA-4 inhibitors is best explained by the presence of CTLA-4 granules in normal pituitary glands of some individuals [60]. The CTLA-4 inhibitors bind to the CTLA-4 granules and elicit type II and type IV immune responses causing T-cell lymphocytic infiltration of the pituitary which ultimately results in destruction of the gland [60], [61]. The median time to the onset of hypophysitis after starting immunotherapy is 2–3 months [62]. It is characterized by the onset of new symptoms of hypopituitarism after starting immunotherapy in the absence of alternate triggers or etiologies. Hypopituitarism presents with symptoms of endocrine dysfunction such as hypercortisolism (91%), hypothyroidism (84%), and hypogonadism (83%) [63]. Red flags include headache, loss of vision, and hypotension [63]. Hypophysitis is treated by cessation of ICI therapy and administration of steroids.

**Fig. 13:**
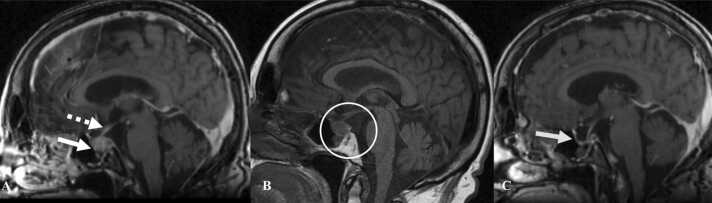
Immune-mediated hypophysitis in a 70-year-old woman with melanoma on Pembrolizumab. A. Contrast-enhanced sagittal MRI image of the brain shows enlargement of the pituitary gland with convex superior margin (arrow) and thickened stalk (dashed white arrow). B. T1W sagittal MRI image of the brain shows absent posterior pituitary bright spot (white circle). C. Contrast-enhanced sagittal MRI image of the brain obtained after Pembrolizumab was stopped and steroid was administered shows significant decrease in the size of pituitary gland with empty sella turcica (arrow).

MRI is the best imaging modality to diagnose hypophysitis. There is diffuse enlargement of the pituitary gland and its stalk with loss of signal intensity in the posterior pituitary on pre-contrast sequences and homogenous enhancement on post-contrast sequences (Fig. 13) [64]. Geographic hypoenhancing lesions are noted in the anterior lobe due to fibrosis of the gland [65]. On PET/CT there is an initial increase in the FDG-uptake of the pituitary gland with subsequent decline in the uptake as the gland undergoes fibrosis [66].

### 6.2 Thyroiditis

The incidence rate of thyroiditis with ICI therapy is 6–20% [1], [67]. Contrary to hypophysitis, the incidence of thyroiditis is higher with PD-1/PD-L1 inhibitors and lower with CTLA-4 inhibitors [68]. The median time to onset of thyrotoxic phase after initiating ICI is 5 weeks and hypothyroid phase is 10 weeks [68]. Thyroid dysfunction occurs in a biphasic pattern with an initial thyrotoxic phase followed by hypothyroidism due destruction of the gland. A prior study by Iyer et al. revealed that combined therapy has a higher incidence rate and shorter time to the onset of thyroiditis (2 weeks) [68]. Thyrotoxicosis is characterized by low thyroid stimulating hormone (TSH) and elevated functional T4 (fT4) while hypothyroidism is characterized by an elevated or normal TSH with low fT4 [68]. It is best treated with steroids and thyroid supplementation [68]. Beta blockers are reserved for symptomatic thyrotoxicosis.

**Fig. 14:**
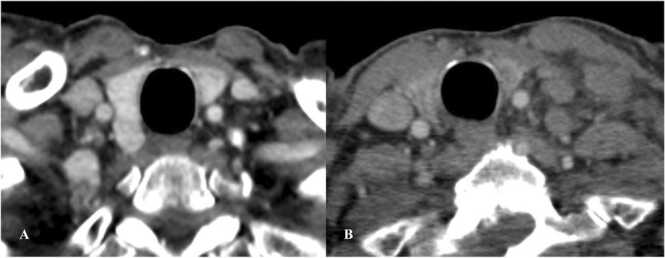
Immune-mediated thyroiditis in a 65-year-old man with lung cancer on Nivolumab. Contrast-enhanced axial chest CT images show A. Normal appearance of the thyroid before starting Nivolumab. B. There is atrophy of the thyroid with decrease in the enhancement after starting Nivolumab consistent with thyroiditis.

On thyroid ultrasound, the gland is heterogenous with low vascularity consistent with thyroiditis [68]. On CT, there is atrophy of the thyroid with decreased enhancement of the gland (Fig. 14) [68]. On PET/CT there is diffuse increase in FDG uptake initially followed by decrease in the uptake with development of fibrosis [68].

### 6.3 Adrenalitis

The incidence of adrenalitis ranges from 0.8-1.6% [40], [69]. The incidence rate is as high as 4–8% when combined CTLA-4 and PD-1/PD-L1 therapy is used [40], [69]. The median time to onset is 2.3–4.5 months [40], [69]. Primary adrenal insufficiency can be identified by electrolyte disturbances and eosinophilia with low serum cortisol along with high adrenocorticotrophin hormone (ACTH) [70]. Adrenal crisis, a life-threatening emergency, is rarely encountered with ICI therapy [1]. When adrenal crisis occurs, there is hypovolemic shock with nausea, vomiting, confusion, or coma [70]. Mild adrenal insufficiency is monitored and ICI therapy is continued [70]. Adrenal crisis is treated with intravenous steroids and fluid replacement [70].

**Fig. 15:**
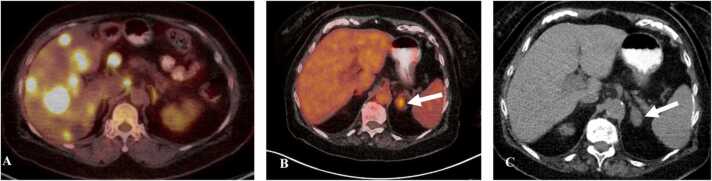
Immune-mediated adrenalitis in a 55-year-old man with melanoma on Pembrolizumab. A. Fused axial PET/CT image shows multiple hepatic metastases and mild FDG avidity in the left adrenal gland. B. Fused axial PET/CT image obtained after 6 months of initiation of Pembrolizumab therapy shows resolution of the hepatic metastases but new FDG uptake in the left adrenal gland (arrow). C. CT correlation shows new smooth enlargement of the left adrenal gland (arrow) seen with adrenalitis.

On CT, adrenalitis is characterized by smooth enlargement of the adrenal glands with homogenous enhancement on post contrast images (Fig. 15) [70]. On PET/CT there can be diffuse FDG uptake in the adrenal glands [70].

## 7 Musculoskeletal irAE

### 7.1 Inflammatory arthritis

The incidence rate of inflammatory arthritis is 5–12% in patients who received ICI therapy [71], [72]. The incidence rate is higher with combined therapy [71]. Median time to onset of arthritis is 3–4 months [72]. Prior studies have revealed that arthralgia or pain in the joints continues to persist after cessation of ICI therapy for 9 months or longer [73]. Symptomatic relief can be obtained by using non-steroidal anti-inflammatory drugs (NSAIDs) or low dose steroids and ICI therapy can be continued [72]. Some patients with more severe symptoms may require cessation of immunotherapy with high dose steroids and disease modifying anti-rheumatoid drugs (DMARDs) [72].

**Fig. 16:**
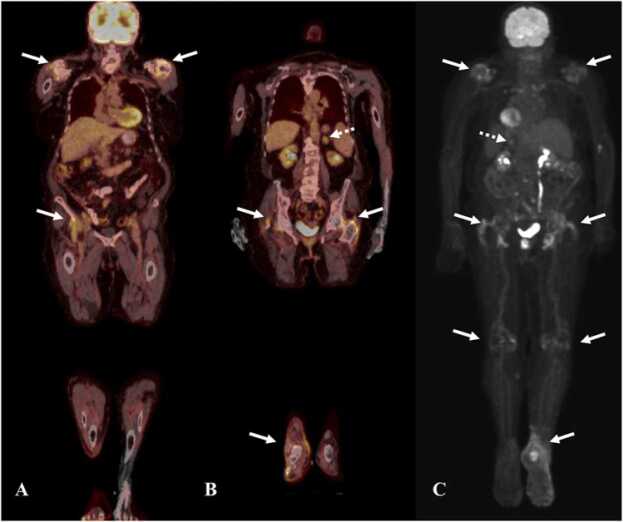
Immune-mediated inflammatory arthritis and adrenalitis in a 63-year-old man with melanoma on Pembrolizumab therapy. A & B. Fused coronal PET/CT images show FDG uptake in bilateral shoulder joints (white arrows), bilateral hip joints and right ankle joint (white arrows), and left adrenal gland (dashed white arrow), C. Reconstructed MIP image shows uptake in bilateral shoulder joints, hip joints, right ankle joint (arrows) and left adrenal gland (dashed white arrow).

Synovitis is best identified on MRI. There is thickening, edema, and hyperenhancement of the synovium with joint effusion [72]. Increased periarticular FDG uptake is seen in synovial tissues on PET/CT [72]. Large joints are more commonly involved in symmetrical fashion [72]. Multiple joints are more involved with the median number of joints involved being four. The most commonly involved joints include the shoulders, knees, ankles, and wrists [72]. On PET/CT there is FDG avidity in the involved joints (Fig. 16).

## 8 CNS irAE

Neurological irAEs are a rare complication of immunotherapy but can result in long-term morbidity. The incidence of neurological irAEs range from 3.8-12% [74]. The incidence is higher when combination therapy is used [74]. A wide-range of neurological manifestations such as myasthenia gravis, Guillain Barre’ syndrome, transverse myelitis, chronic inflammatory demyelinating polyneuropathy, meningitis, posterior reversible encephalopathy syndrome, and encephalopathy can be seen with immunotherapy related toxicity [75].

### 8.1 Toxic encephalopathy

**Fig. 17:**
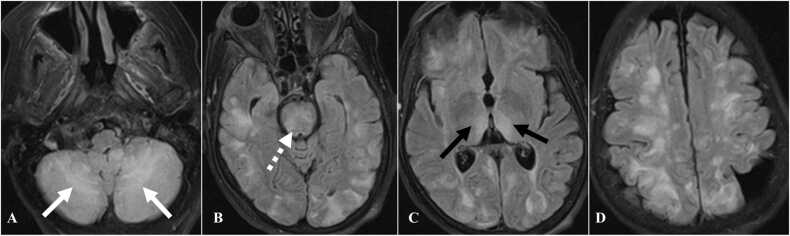
Immune mediated toxic encephalopathy in a 69-year-old woman with lung cancer on Pembrolizumab therapy. A to D. Axial FLAIR images of MRI of the brain demonstrate subcortical and periventricular hyperintensities. Also seen are hyperintense areas in the cerebellum (white arrow), pons (dashed white arrow), and thalamus (black arrows).

Patients who develop toxic encephalopathy due to immunotherapy can present with confusion, headache, vomiting, disorientation, and confabulation. CT may not show any findings in acute phase. MRI is the imaging modality of choice. On fluid-attenuation inversion recovery (FLAIR) sequence there is hyperintensity in subcortical and periventricular white matter, thalamus, corpus callosum, pons, and cerebellum (Fig. 17) [76].

## 9 Multiorgan irAEs

Multiorgan irAEs occur less frequently and range from 5.4-9.3% [77]. Patients with multiorgan irAEs have better response and improved survival compared to patients with single irAE. Prior studies have revealed that patients with multiorgan irAEs have 42% response rate and 53% reduced likelihood of death [78].

## 10 Conclusion

ICI therapy brought a paradigm shift in the treatment of solid and hematological malignancies. Though ICIs improve patient outcomes, they can be associated with adverse events in the form of irAEs. Patients who develop irAEs have better outcomes and improved survival than patients who do not develop irAEs. Therefore, it is vital to identify irAEs early and start mitigation measures immediately to continue beneficial ICI therapy without interruption. Radiologists must be aware of the imaging features of irAEs and alert the oncology team as soon as they identify a complication, albeit good prognostication, of ICI therapy.

## Funding

None.

## CRediT authorship contribution statement

**Babina Gosangi**: Conceptualization, Figures, Writing – original draft, Writing – review & editing. **Lacey McIntosh**: Conceptualization, Figures, Writing – review & editing. **Abhishek Keraliya**: Figures, Writing – review & editing. **David V.K. Irugu**: Figures, Writing – review & editing. **Akshay Baheti**: Writing – review & editing. **Ashish Khandelwal**: Writing – review & editing. **Richard Thomas**: Writing – review & editing. **Marta Braschi Amirfarzan**: Conceptualization, Figures, Writing – review & editing.

## Conflict of Interest

None.
